# Increasing levels of *Parasutterella* in the gut microbiome correlate with improving low-density lipoprotein levels in healthy adults consuming resistant potato starch during a randomised trial

**DOI:** 10.1186/s40795-020-00398-9

**Published:** 2020-12-11

**Authors:** Jason R. Bush, Michelle J. Alfa

**Affiliations:** 1 MSP Starch Products Inc., Carberry, MB Canada; 2 Department of Biology, Brandon University, Brandon, MB Canada; 3 Department of Medical Microbiology, University of Manitoba, Winnipeg, MB Canada

**Keywords:** *Parasutterella*, Proteobacteria, Potato, Resistant starch, LDL, Cholesterol

## Abstract

**Background:**

Prebiotics, defined as a substrate that is selectively utilized by host microorganisms conferring a health benefit, present a potential option to optimize gut microbiome health. Elucidating the relationship between specific intestinal bacteria, prebiotic intake, and the health of the host remains a primary microbiome research goal.

**Objective:**

To assess the correlations between gut microbiota, serum health parameters, and prebiotic consumption in healthy adults.

**Methods:**

We performed ad hoc exploratory analysis of changes in abundance of genera in the gut microbiome of 75 participants from a randomized, placebo-controlled clinical trial that evaluated the effects of resistant potato starch (RPS; MSPrebiotic®, *N* = 38) intervention versus a fully digestible placebo (*N* = 37) for which primary and secondary outcomes have previously been published. Pearson correlation analysis was used to identify relationships between health parameters (ie. blood glucose and lipids) and populations of gut bacteria.

**Results:**

Abundance of *Parasutterella* (phylum Proteobacteria) tended to increase in the gut microbiome of individuals consuming RPS and those increases in *Parasutterella* were correlated with reductions in low-density lipoprotein (LDL) levels in participants consuming RPS but not placebo. Segregating RPS-consuming individuals whose LDL levels decreased (ie “Responders”) from those who did not (ie. “Non-Responders”) revealed that LDL Responders had significantly higher levels of *Parasutterella* both at baseline and after 12 weeks of consuming RPS.

**Conclusion:**

Our analyses suggest that RPS may help improve LDL levels depending upon the levels of *Parasutterella* in an individual’s gut microbiome.

**Trial registration:**

This study protocol was reviewed and approved by Health Canada (Submission #188517; “Notice of Authorization” dated 06/05/13) and registered as NCT01977183 (10/11/13) listed on NIH website: ClinicalTrials.gov. Data generated in this study have been submitted to NCBI (http://www.ncbi.nlm.nih.gov/bioproject/381931).

**Funding:**

MSP Starch Products Inc.

## Background

The ecosystem of microbes in the human intestines, often referred to as the gut microbiome, influences a wide range of physiological processes and methods to manipulate these connections are actively being investigated [1]. Prebiotics, defined as “a substrate that is selectively utilized by host microorganisms conferring a health benefit”, stimulate the growth of certain populations of beneficial microbes and therefore offer a strategy to favorably alter the gut microbiome [2]. Prebiotic consumption can positively affect the physiology of the host as well as the microbiome [3, 4], motivating further investigation into the potential health benefits of prebiotics. While the relationships between specific gut microorganisms, dietary intake, and host health outcomes are broadly applicable in principle, dietary interventions to promote host health outcomes may have varying effects depending on the baseline composition of the host’s gut microbiome [5]. This reflects both the promise and the challenge of capitalizing on personalized nutrition.

We previously described a clinical trial examining the effects of the MSPrebiotic® (also marketed as the ingredient Solnul™) resistant potato starch (RPS) on the microbiome and various health parameters in people 30–50 years old and those 70 years of age or older [3, 4]. We demonstrated that RPS led to significant increases in *Bifidobacterium*, reductions in *Escherichia*, enhanced butyrate production [4], and improvements in blood glucose, insulin, and insulin resistance [3] that were correlated with reductions in the abundance of *Sporacetigenium* [6].

The connections between starch-fermenting *Bifidobacterium*, butyrate-producing members of the phylum Firmicutes, and health outcomes have been well-documented [7] but little research has been done to establish connections between other, more obscure bacteria in the gut microbiome. Furthermore, the role of Proteobacteria in the gut microbiome remains ambiguous, with evidence supporting both healthy [8] and pathogenic relationships [9]. Here, we report that levels of *Parasutterella* (phylum Proteobacteria), a core member of the gut microbiota [10], were increased in a subset of people consuming RPS. The objective of this study was to explore connections between *Parasutterella* and various metabolic markers in RPS-consuming individuals to determine the relevance of this gut microbiome alteration.

## Methods

### Clinical study, sample collection, and processing

**Fig. 1:**
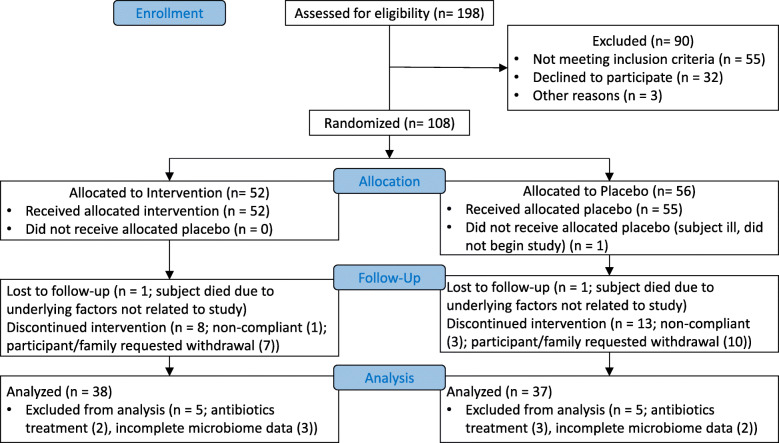
CONSORT Flow Diagram. Number of participants analyzed; RPS *n* = 38, placebo *n* = 37, unless otherwise specified

The primary and secondary endpoints of this prospective, randomized, blinded, placebo-controlled study have been previously described in detail [3, 4]. Study design, enrollment, analysis, and interpretation follow CONSORT guidelines, and a full study protocol is available upon request. Enrollment began in September 2013 and was completed in May 2015. Adults (aged 30–50 years or aged 70 years or older; 75 analyzed; Fig. 1) were recruited in Winnipeg, MB, Canada. Participants (or an authorized third party) provided written informed consent in accordance with the Declaration of Helsinki and in compliance with the University of Manitoba guidelines. Participants were advised that they could withdraw from the study at any time without negative consequences. Exclusion criteria included: Pregnancy, diagnosis with Crohn’s disease or other inflammatory bowel disease, systemic lupus erythematosus, prediabetes or diabetes, thyroid disease, renal disease, hepatic disease, dysphagic individuals or those who previously had gastrointestinal surgery (intestinal resection, gastric bypass or colorectal surgery), those on cancer chemotherapy, consuming probiotics or fermented foods (ie. yogurt), on antibiotics at recruitment or within the 5 previous weeks, subjects using additional fiber supplements, and individuals on digestants, emetics, anti-emetics, medications for acid peptic disorders, or antacids. Participants consumed 30 g of placebo (digestible corn starch; Amioca TF, Ingredion, Brampton, ON, Canada) daily for 2 weeks before randomization to placebo (*N* = 37) or resistant potato starch (RPS as MSPrebiotic®; MSP Starch Products Inc., Carberry, MB, Canada; *N* = 38) arms (Fig. 1). RPS and placebo products were comparable in appearance and organoleptic properties. Participants were assigned to placebo or study product by the study coordinator based on a list of computer-generated randomized numbers, and then consumed 30 g of placebo or RPS daily for 12 weeks (14 weeks total). Stool and fasting blood samples were collected at baseline and 14 weeks. Blood glucose and lipid (total cholesterol, triglycerides, low-density lipoprotein, high-density lipoprotein) levels were determined by Diagnostic Services Manitoba (Winnipeg, MB) and insulin levels by LipoScience Inc. (Raleigh, NC). Gut microbiome analysis was performed by 16S rRNA sequencing on the Illumina MiSeq platform and alignment as previously described [4, 11, 12]. The data from all participants (regardless of age) was pooled for this analysis. Care providers, trial participants, laboratory testing personnel, and data analysts were blinded to which arm participants were assigned.

### Amino acid analysis

Samples of RPS (MSP Starch Products Inc., Carberry, MB, Canada) and green banana starch (Natural Evolution, Walkamin, QLD, Australia) were sent to Bureau Veritas Laboratories (Mississauga, ON, Canada) and amino acid contents were determined using AOAC 982.30 methods.

### Statistical analysis

Baseline values were subtracted from week 14 values and expressed as a change in percent (blood lipid, glucose, and insulin levels) or a change in relative abundance (bacteria) for each participant. Pearson’s correlation coefficients (*r*) for changes in *Parasutterella* and changes in blood chemistry were calculated and *p* values determined using Excel (Microsoft, Redmond, WA), as were Student’s one-way t-Test calculations. We employed the Benjamini-Hochberg procedure [13] at a false discovery rate (FDR; *q*) of 0.1 to control for multiple testing during correlation analysis. The critical values for each parameter were generated by dividing the *p* value rank (*i*) by the total number of parameters analyzed (*m*), then multiplying this quotient by the FDR (*q*). Microbiome variability was expressed as the standard error of the mean (SEM) while blood chemistry variability was expressed as the standard deviation (SD), with *p* < 0.05 considered statistically significant.

## Results

**Fig. 2:**
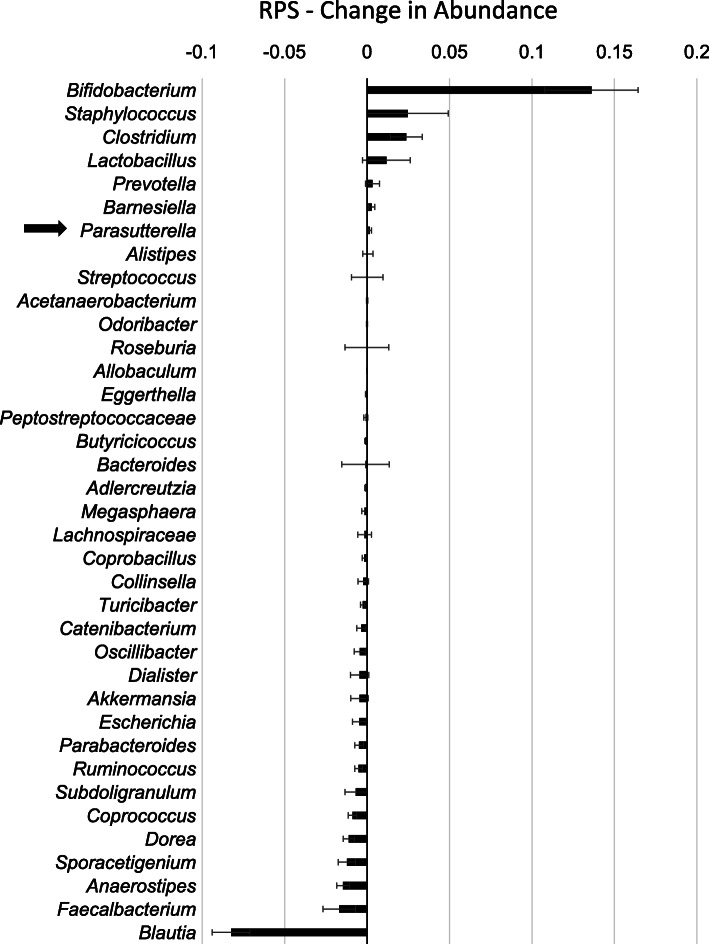
Mean change (+/− SEM) in relative abundance for each genus discretely identified in individuals consuming RPS for 12 weeks. *Parasutterella*, indicated by the black arrow, was the only genus in phylum Proteobacteria to increase in response to RPS

**Fig. 3:**
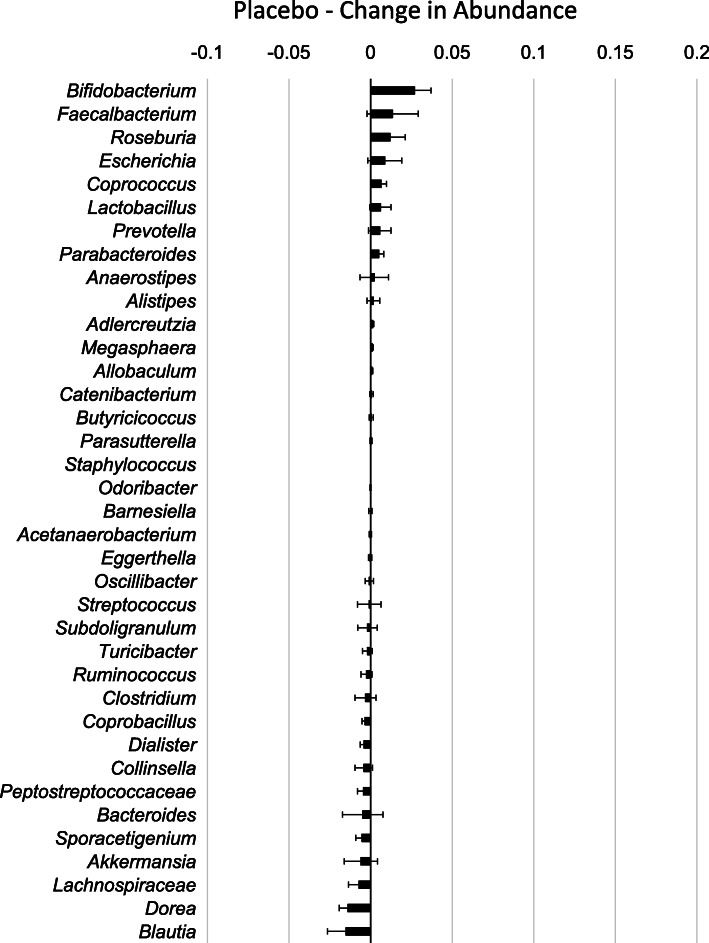
Mean change (+/− SEM) in relative abundance for each genus discretely identified in individuals consuming placebo for 12 weeks

**Fig. 4:**
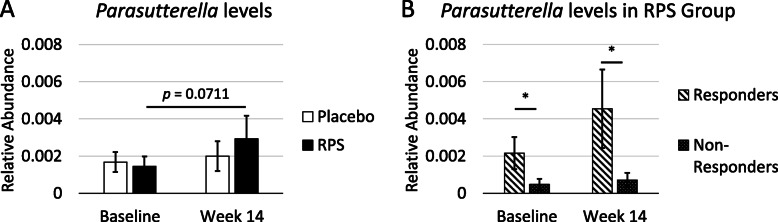
**a** RPS consumption tended to increase mean levels of *Parasutterella* by two-fold (*p* = 0.0711) while *Parasutterella* levels were unchanged in those consuming placebo (+/− SEM, *p* = 0.291). **b** Segregation of the RPS group into those who displayed a decrease in LDL levels (Responders) and those whose levels increased or remained the same (Non-Responders) revealed that mean *Parasutterella* levels were significantly higher in Responders at both baseline and week 14 (+/− SEM). *; *p* < 0.05

RPS consumption led to increases in some genera of bacteria and decreases in others [4, 6] (Fig. 2) while changes in response to placebo were minor (Fig. 3). The effects on *Bifidobacterium* have previously been described [4] and here we find that RPS significantly increased *Clostridium* (*p* = 0.020716) and decreased *Blautia* (*p* < 0.000001), but had no significant effects on *Staphylococcus* (*p* = 0.323284) or *Faecalbacterium* (*p* = 0.094695). We previously reported that RPS consumption led to reduced *Escherichia coli* levels [4] but *Parasutterella* was the only genus belonging to phylum Proteobacteria that increased in those consuming RPS (Fig. 2). This two-fold increase trended towards significance in RPS consumers (Fig. 4a, *p* = 0.0711) but placebo consumption had no effect on *Parasutterella* levels (Fig. 4a, *p* = 0.291).

We previously demonstrated that decreases in *Sporacetigenium* were correlated with improvements in blood glucose and insulin in people consuming RPS [6], so we investigated whether increases in *Parasutterella* in response to RPS consumption were correlated with markers of cardiovascular and/or metabolic health. Pearson correlation coefficients (*r*) and *p* values were calculated for changes in *Parasutterella* and changes in total cholesterol, triglycerides, low-density lipoprotein (LDL), high-density lipoprotein (HDL), blood glucose, and insulin levels. To control for multiple testing, the *p* values were then compared for significance using the Benjamini-Hochberg method (Table 1). Changes in *Parasutterella* were significantly inversely correlated with changes in LDL levels (i.e. as *Parasutterella* increased, LDL levels decreased) in individuals consuming RPS (*r* = − 0.400461; *p* = 0.01284) but not with the other parameters. Notably, changes in *Parasutterella* were not significantly correlated with LDL levels in the placebo group (*r* = 0.230647; *p* = 0.1697).

**Table 1:** Correlations between the change in abundance of *Parasutterella* and changes in total cholesterol, triglycerides, low-density lipoprotein (LDL), high-density lipoprotein (HDL), blood glucose, and insulin levels in individuals consuming RPS

Health Parameter	***r***	***p*** value	Rank (***i***)	Critical Value ([***i***/***m***]*q)
**LDL**	**−0.40046151**	**0.01284**	**1**	**0.016667**
Blood Glucose	0.256738899	0.119769	2	0.033333
Cholesterol	−0.18932218	0.255775	3	0.05
Insulin*	0.130393557	0.448782	4	0.066667
HDL	−0.06547371	0.698229	5	0.083333
Triglycerides	0.037206775	0.824522	6	0.1

The Benjamini-Hochberg method controls for false discovery of significant correlations [13]. Results are rank ordered based on *p* value, and the *p* value is compared to the critical value ([*i*/*m*]*q; FDR (*q*) = 0.1) beginning with the lowest ranking parameter (Triglycerides). The first correlation with a *p* value lower than the critical value (LDL) and any higher-ranking correlations are considered significant (bold). Positive Pearson correlation coefficient (*r*) values indicate positive correlations and negative *r* values indicate negative correlations. *N* = 38 except for Insulin*, where *N* = 36 due to missing insulin measurements

Despite the correlation between changes in *Parasutterella* and changes in LDL levels, RPS consumption did not lead to overall changes in LDL compared to placebo-consuming controls [4]. *Parasutterella* is found in low abundance in the gut microbiome [10], suggesting that a minimum threshold may be required for RPS-mediated effects on LDL. We therefore evaluated whether baseline *Parasutterella* levels were higher in RPS consumers who experienced a decrease in LDL levels (Responders) compared to those in which LDL levels increased or were unchanged (Non-Responders). Consistent with this hypothesis, we found that baseline *Parasutterella* levels were significantly more abundant in Responders compared to Non-Responders (Fig. 4b, *p* = 0.03892). This difference between Responders and Non-Responders was more pronounced at the end of the trial (Fig. 4b, *p* = 0.04401), although Responders’ Week 14 levels were not significantly different from those at baseline (*p* = 0.15267).

**Fig. 5:**
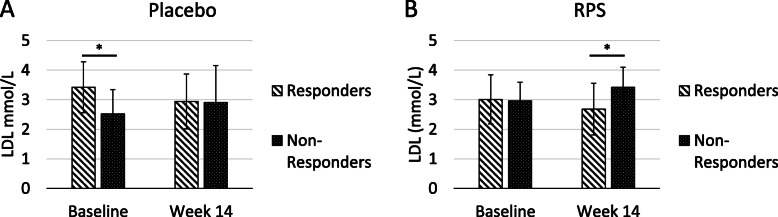
**a** Baseline LDL levels were significantly different between Responders and Non-Responders in the Placebo group (*p* = 0.00245) but indistinguishable at Week 14 (+/− SD; *p* = 0.91978). **b** Baseline LDL levels were indistinguishable between Responders and Non-Responders in the RPS group (*p* = 0.85119) but were significant different at Week 14 (+/− SD; *p* = 0.00814). *; *p* < 0.05

It is possible that LDL changes observed are independent of RPS supplementation. For example, Responders may have high baseline LDL levels that decrease over time and Non-Responders may have a low baseline LDL levels that rise over time. To address this possibility, we segregated participants in the Placebo group into Responders (LDL levels decrease) and Non-Responders (LDL levels increase or are unchanged) as described for the RPS group. We found that baseline LDL levels were significantly different between Responders and Non-responders in the placebo group (*p* = 0.00245) but nearly identical at Week 14 (*p* = 0.91978; Fig. 5a). Thus, the LDL responses in the placebo group were due to differences in baseline levels prior to intervention. Conversely, baseline LDL levels were nearly identical between Responders and Non-Responders in the RPS group (*p* = 0.85119) but significantly different at Week 14 (*p* = 0.00814; Fig. 5b), which indicates the LDL responses in the RPS group are due to the intervention. Taken together, these data suggest that RPS consumption leads to reduced LDL levels in individuals with above-threshold levels of *Parasutterella* and that serum LDL response is inversely proportional to *Parasutterella* changes in the stool.

Characterization of *Parasutterella* revealed a genomic signature consistent with an inability to ferment starch, and in vitro culture assays demonstrated preferential catabolism of non-essential amino acids and growth on non-essential amino acid substrates such as L-asparagine and L-aspartic acid [10]. Given that potato starches have previously been demonstrated to contain these amino acids [14], we assessed whether RPS similarly contained amino acids that could support the growth of *Parasutterella*. Unlike green banana starch, RPS contained no amino acids except trace amounts of tryptophan (Table 2). This suggests that increased levels of *Parasutterella* in the gut microbiome in response to RPS supplementation is not due to L-asparagine and L-aspartic acid derived from RPS [10].

**Table 2:** Amino acid abundance in resistant starch from potato and green banana sources

Amino Acid	Resistant Potato Starch	Green Banana Starch
Alanine	ND	0.16
Arginine	ND	0.21
Aspartic Acid	ND	0.59
Cystine	ND	ND
Glutamic Acid	ND	0.56
Glycine	ND	0.14
Histidine	ND	0.13
Isoleucine	ND	0.10
Leucine	ND	0.19
Lysine	ND	0.14
Methionine	ND	0.04
Phenylalanine	ND	0.13
Proline	ND	0.15
Serine	ND	0.14
Taurine	ND	0.05
Threonine	ND	0.11
Tryptophan	0.05	0.08
Tyrosine	ND	0.08
Valine	ND	0.14
**Total**	**0.05**	**3.14**

Amino acids measured using the AOAC 982.30 methodology. All values are reported as g/100 g. The reportable detection limit for each amino acid is 0.01 g/100 g. *ND* Not detected

## Discussion

While species belonging to the genera *Bifidobacterium* and *Ruminococcus* are generally recognized as the only primary degraders of resistant starch [15], consumption of resistant potato starch (RPS) led to significant changes in several different genera, including those belonging to phylum Proteobacteria. Members of the gut microbiome belonging to Proteobacteria are typically considered to be undesirable, and are associated with infectious diarrhea, elevated inflammation, increased permeability of the gut wall and the production of harmful metabolites [16]. Here, we sought to understand subtle changes in non-primary resistant starch degraders and report a correlation between increasing levels of *Parasutterella* (phylum Proteobacteria), a core member of the gut microbiota [10], and decreasing levels of LDL, an important risk factor for cardiovascular disease. Notably, the linear correlation between increases in *Parasutterella* and decreases in LDL facilitates integration into gut microbiome testing platforms, such that interventions (such as prebiotic or probiotic supplementation) that increase *Parasutterella* will be predicted to similarly reduce LDL levels in a proportional manner. While the effect of RPS on LDL cholesterol was modest (Fig. 5b; mean change - 0.33 mmol/L in Responders) compared to cholesterol lowering medications [17], RPS supplementation may help reduce LDL levels in combination with other therapies. The evaluation of *Parasutterella* levels in the gut microbiome to predict a person’s response to RPS is consistent with a personalized approach to medicine and speaks to growing appreciation for the role that differences in gut microbiome composition play in shaping human health.

*Parasutterella* is an anaerobic, asaccharolytic Gram-negative, non-spore-forming coccobacillus, that was originally described based on a strain isolated from a fecal sample from a healthy Japanese male [18]. Deep sea water (DSW) is one of several dietary supplements that tend to increase *Parasutterella* levels [19–22]. Using a diet-induced hamster model of hypercholesterolemia, Lin and colleagues demonstrated that DSW also led to significant reductions in triglycerides, LDL, and total cholesterol, although *Bacteroidetes* was the only bacterial population significantly correlated with serum cholesterol and LDL in response to the high cholesterol diet [20]. Increases in *Parasutterella* in response to GOS supplementation in mice were associated with significant reductions in triglycerides but not LDL levels [23]. *Parasutterella* levels were inversely correlated with fat consumption but not total energy intake in obese people [24].

Introduction of *Parasutterella* into normal mice led to reductions in cecal levels of cholic acid, taurocholic acid, taurodeoxycholic acid, 7-ketodeoxycholic acid (or isomers), and glycolithocholic acid [10]. Additionally, there were concomitant increases in taurine and changes in bile acid metabolism that were consistent with bacteria-mediated deconjugation of primary bile acids [10]. Furthermore, the same study demonstrated changes in farnesoid X receptor (FXR)-dependent gene expression, including increases in *Cyp7a1*, suggesting enhanced bile acid synthesis [10]. Mushroom polysaccharide supplementation of a high fat diet led to similar changes in gene expression, along with increases in *Parasutterella* that were correlated with reductions in serum lipids [25]. While total cholesterol levels were decreased, although not significantly, by the introduction of *Parasutterella*, LDL levels were not measured [10], suggesting that the mechanisms documented in mice could be acting similarly in humans consuming RPS, specifically those with above-threshold levels of *Parasutterella*. Furthermore, although stable colonization of *Parasutterella* in the gastrointestinal tract occurred rapidly in mice [10], it is possible that the mean changes in bile acid abundance, FXR-dependent gene expression, and the effects on cholesterol are driven by mice in which higher levels of *Parasutterella* engrafted to the gut microbiome, consistent with the data presented here for humans.

Despite potatoes having been identified as dietary sources of asparagine and aspartic acid [14], our analysis reveals that all amino acids, except for a trace amount of tryptophan, are absent in RPS. Thus, it is unclear from our data how RPS supports the growth of *Parasutterella*. It is intriguing to note that *Parasutterella* was identified as part of a co-abundance response group that increased in response to chemically modified resistant starches (Type 4) in people [26]. This suggests that resistant starch may generally support the growth of *Parasutterella*, through cross-feeding or some other indirect mechanism(s). Indirect growth via complex ecological interactions could explain why RPS consumption stimulated growth of *Parasutterella* in some people and not others. Furthermore, Fig. 2 demonstrates that there are a number of significant microbial shifts within the gut microbiome that occur in humans consuming RPS (e.g. *Bifidobacteria* and *Clostridium* increase while *Blautia* decrease). This suggests there may be a “co-abundance” type response in humans related to RPS consumption, though the interactions between Parasutterella and these genera remain to be elucidated. This supports Marchesi and others’ statement [27]: “*However, as we learn more about the ecology of the gut microbiota it is becoming clear that the prebiotic concept has tapped into the underlying fabric of the gut microbiota as a primarily saccharolytic and fermentative microbes community evolved to work in partnership with its host’s digestive system to derive energy and carbon from complex plant polysaccharides which would otherwise be lost in faeces.*”

Limitations of our study include the large dose (30 g/day), the relatively small study population, and the use of age brackets within that population. Furthermore, statistical analysis of *Parasutterella* levels was hampered by the low relative abundance of this genus (mean levels < 0.4%). Future studies examining the response of *Parasutterella* to lower doses of RPS in a larger, general population are warranted.

## Conclusions

We propose that the effect of *Parasutterella* on the host’s physiology is dependent upon a variety of factors, including prebiotic consumption, the baseline level of this organism and the co-abundance response of the host’s gut microbiome. Further research is required to identify these factors and the mechanisms by which they interact to influence cholesterol homeostasis.

## Data Availability

Data generated have been submitted to NCBI (http://www.ncbi.nlm.nih.gov/bioproject/381931).

## Abbreviations

DSW: Deep sea water
FDR: False discovery rate
FXR: Farnesoid X receptor
HDL: High density lipoprotein
LDL: Low density lipoprotein
ND: Not detected
RPS: Resistant potato starch
SD: Standard deviation
SEM: Standard error of the mean
